# Increased concentration of serum soluble LAG3 in systemic lupus erythematosus

**DOI:** 10.1186/ar3617

**Published:** 2012-02-09

**Authors:** Seri Yu, Keishi Fujio, Kazuyoshi Ishigaki, Hirofumi Shoda, Tomohisa Okamura, Tanita Noor, Shuji Sumitomo, Kazuhiko Yamamoto

**Affiliations:** 1 Department of Allergy and Rheumatology, Graduate School of Medicine, The University of Tokyo, 113-0033, Japan

## Background

In systemic lupus erythematosus (SLE), type I interferon and plasmacytoid DCs (pDCs) are supposed to play important roles. However, there are few evidences for pDCs activation in SLE. Murine pDCs are reported to produce soluble LAG3 (sLAG3) upon activation and pDCs are responsible for most of sLAG3 in mice serum [1]. Therefore, serum sLAG3 concentration was examined in SLE and other autoimmune diseases.

## Materials and methods

This study enrolled 45 SLE patients who met ACR criteiria. Disease activity was rated using a SLE disease activity index (SLEDAI). sLAG3 concentrations were measured by a quantitative sandwich enzyme immunoassay [2].

## Results

The ratio of sLAG3 concentration in SLE to control was 3.10+/-1.05, PM/DM to control was 1.04+/-0.08, and RA to control was 0.77+/-0.14. In addition, sLAG3 concentrations showed a significant correlation with SLEDAI. Interestingly, elevation of sLAG3 was observed even in patients with SLEDAI = 0. These results suggested that sLAG3 could be a specific and novel marker for SLE.

## Conclusions

sLAG3 can be a novel marker for SLE. sLAG3 in sera of SLE patient may reflect the activation of pDCs. Because sLAG3 shows adjuvant effect when combined with active immunization [3], sLAG3 may contribute to the exacerbation of lupus. The association between elevated sLAG3, type I interferon signature and activation of pDCs should be investigated further.

**Figure 1:**
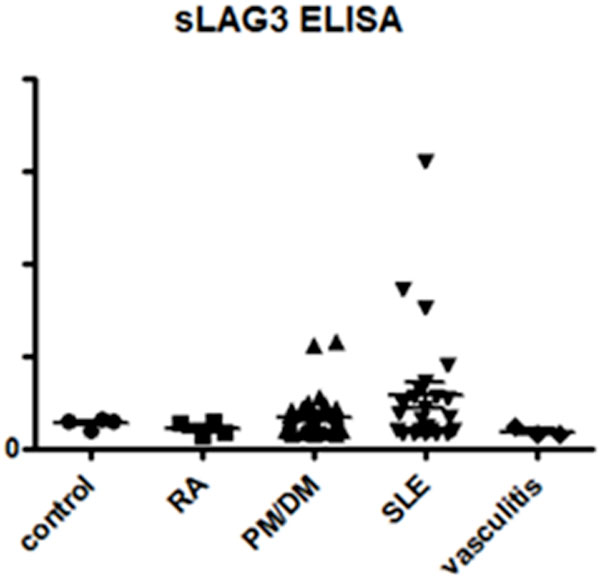
**sLAG3 concentrations in SLE and other autoimmune diseases measured by ELISA**.

